# Bilateral Peripheral Facial Nerve Neuropathy as the Presenting Feature of Primary Sjögren Syndrome: A Case Report

**DOI:** 10.1002/ccr3.70940

**Published:** 2025-09-28

**Authors:** Ryosuke Hanaoka

**Affiliations:** ^1^ Department of Rheumatology and Internal Medicine Kamitsuga General Hospital Kanuma Japan

**Keywords:** bilateral facial palsy, extra‐glandular involvement, facial nerve, peripheral neuropathy, Sjogren syndrome

## Abstract

Bilateral facial nerve paralysis can be the presenting feature of primary Sjögren syndrome. Minor salivary gland biopsy and anti‐SS‐A antibody testing are essential for diagnosis when sicca symptoms are mild, as this rare manifestation may be underdiagnosed without histopathological investigation.

## Graphical abstract

Minor salivary gland biopsy demonstrating lymphocytic infiltration with Greenspan's grade 4 foci, supporting the diagnosis of Sjögren's syndrome in a patient presenting with bilateral peripheral facial nerve palsy.

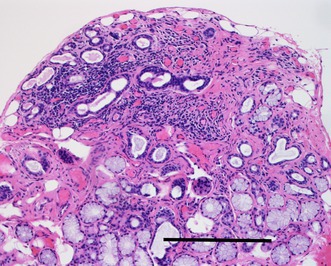

## 1 Introduction

Primary Sjogren syndrome (pSS) is a systemic inflammatory autoimmune disease of unknown etiology that is characterized by salivary and lacrimal gland dysfunction due to infiltration of autoreactive lymphocytes [1]. Patients with pSS can display various types of extra‐glandular involvement due to autoimmunity, including peripheral neuropathy, bronchitis, pneumonitis, and interstitial nephritis [2]. According to different study cohorts, neurological manifestations can occur in 18%–45% of pSS patients, with the peripheral neuropathy being the most frequent [3].

Cranial neuropathy is not so common in peripheral neuropathy complicated by pSS [4]. While the trigeminal nerve is affected most often, facial nerve neuropathy is not common, and those of bilateral involvement are further rare in cranial neuropathy in pSS [4]. Here, we describe a rare case of pSS that presented as peripheral neuropathy in the bilateral facial nerves.

## 2 Case History/Examination

An 83‐year‐old woman with a past medical history of autoimmune hepatitis, diagnosed 9 years earlier and successfully treated with prednisolone, was admitted to our hospital complaining of difficulty closing her right eye. Prednisolone had been tapered and discontinued 8 years earlier, and she had not received any immunosuppressive therapy since that time. She had no history of ocular or oral dryness. Four weeks before this admission, she had visited a neurologist after noticing disordered closure of the left eye and the mouth, and was diagnosed with left peripheral facial nerve paralysis. The neurologist prescribed 30 mg daily of prednisolone and 1000 mg daily of acyclovir. The prednisolone was tapered at a 2‐day interval and discontinued after 8 days. She felt that her facial palsy had partially improved. When she presented at our hospital with the right eye closure disorder, she was admitted for further investigation and treatment.

Her physician had prescribed 5 mg daily of amlodipine, 0.5 μg daily of alfacalcidol, 35 mg weekly of alendronate, 90 mg daily of bethanechol chloride, and 90 mg daily of urapidil for hypertension, osteoporosis, and urinary disturbance.

On admission, her heart rate was 77 bpm, blood pressure was 138/65 mmHg, body temperature was 36.8°C, and oxygen saturation was 99%. There was bilateral absence of wrinkles on the skin of the forehead and narrowing of the palpebral fissures. Mild bilateral hearing loss in the high frequency range was considered mild presbycusis. There were no blisters or redness on the skin of the face or in the ear canals; there was drooping of the corners of the mouth, and the cervical lymph nodes were not palpable. Cranial nerve examinations were otherwise normal, and the patient was diagnosed with bilateral peripheral facial nerve paralysis.

Unstimulated saliva secretion was decreased to 0.5 mL in 15 min, and the gum test to 0.8 mL in 10 min. Dry eye findings were confirmed in the ophthalmological department of our hospital. Schirmer's test I measure was 7 mm on the right side and 11 mm wetting per 5 min on the left side. Corneal fluorescent staining was positive.

## 3 Differential Diagnosis, Investigations, and Treatment

Laboratory values were as follows: angiotensin‐converting enzyme, 15.4 IU/L; IgG, 1237 mg/dL; IgA, 410 mg/dL; IgM, 34 mg/dL; C3, 120 mg/dL; C4, 29 mg/dL; anti‐nuclear antibody negative; anti‐SS‐A antibody, 551 U/mL; anti‐SS‐B antibody, < 0.1 U/mL; MPO‐ANCA, < 0.5 U/mL; PR3‐ANCA, < 0.5 U/mL; and anti‐acetylcholine receptor antibody negative. There was no evidence of antibodies to *Borrelia burgdorferi* . Erythrocyte sedimentation rate and serum C‐reactive protein were within normal ranges. Immunoglobulin M (IgM) antibody for varicella zoster virus and IgM antibody for herpes simplex virus were negative. The protein level and cell count in cerebrospinal fluid were within normal ranges. MRI of the brain revealed no significant abnormal signal intensities and excluded lesions in the 5th, 7th, and 8th cranial nerves. Chest CT revealed no significant abnormality.

**FIGURE 1:**
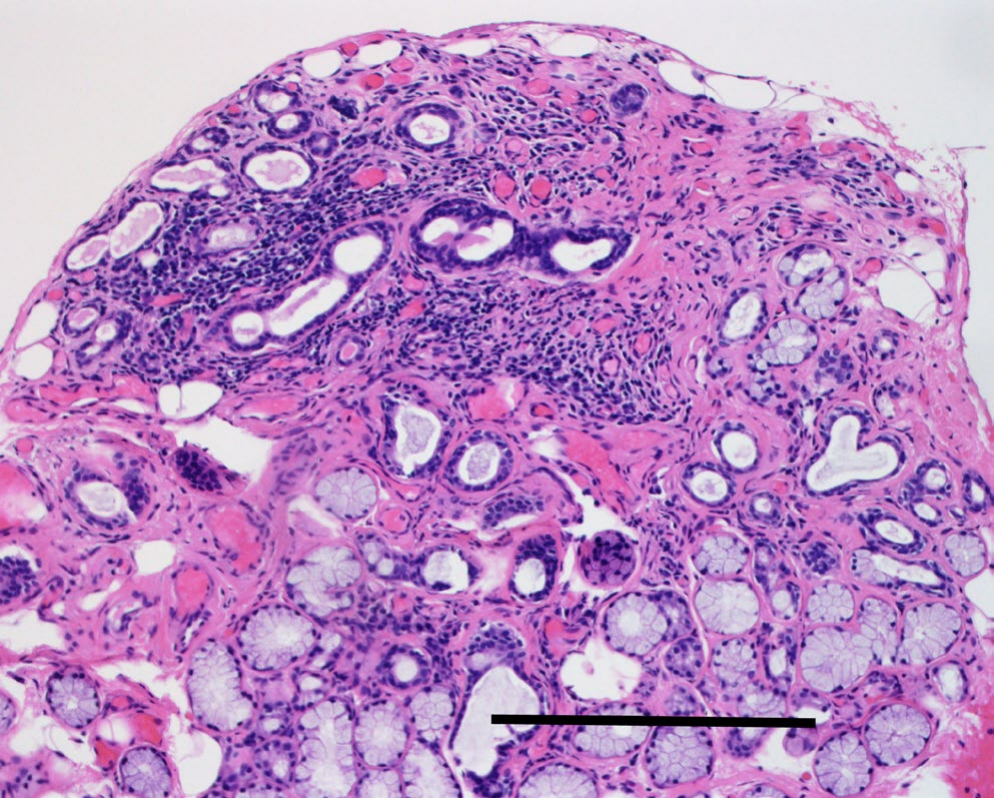
Histopathological features of minor salivary gland biopsy tissue. Scale bar = 500 μm. There is mild to moderate atrophy of serous and mucous glands with marked infiltration of lymphocytes around the salivary duct. Two foci can be seen, each consisted of > 50 lymphocytes within an area of 3 mm^2^. The foci were Greenspan's grade 4.

We performed minor salivary gland biopsy to identify the cause of the elevated anti‐SS‐A antibody level. Histopathological examination of the biopsy tissue revealed mild to moderate atrophy of serous and mucous glands with marked infiltration of small round cells and lymphocytes around the salivary duct, and inconspicuous fibrosis. There were two foci of > 50 lymphocytes within 3 mm^2^ (Figure 1) that were classified as grade 4 on the Greenspan system. According to the 2016 ACR‐EULAR Classification Criteria for primary Sjögren's syndrome, we diagnosed her symptoms as pSS fulfilled by the following: anti‐SSA(Ro) antibody positivity and focal lymphocytic sialadenitis with a focus score ≥ 1 foci/mm^2^, an abnormal ocular staining score ≥ 5, and an unstimulated salivary flow rate ≤ 0.1 mL/min [5].

We prescribed 30 mg daily of prednisolone, which was tapered every 2 days, and then switched to 10 mg every other day of prednisolone and 3 mg daily of tacrolimus.

## 4 Conclusion and Results (Outcome and Follow‐Up)

The bilateral peripheral facial nerve paralysis ameliorated gradually and had almost disappeared after 3 months of treatment with prednisolone and tacrolimus. This case demonstrates that bilateral facial nerve neuropathy can be the presenting feature of primary Sjögren syndrome and that minor salivary gland biopsy combined with serological testing is crucial for accurate diagnosis when sicca symptoms are mild.

## 5 Discussion

We described a rare case of peripheral neuropathy of the bilateral facial nerves with coexistence of pSS. It is reported that peripheral neuropathy as extra‐glandular manifestation is seen in approximately 25% of patients with pSS [6] and is classified as polyneuropathy, mononeuropathy, mononeuropathy multiplex, polyradiculopathy, or cranial neuropathy according to the spatial distribution [3, 4]. Mori et al. reported that cranial neuropathy was seen in 21.7% of patients with peripheral neuropathy complicated by pSS, most‐commonly manifested as trigeminal nerve neuropathy (16.3%), and facial nerve neuropathy (2.2%) was less common and manifested as one of the involvements of multiple cranial nerves neuropathy [4]. It is assumed that bilateral facial nerve neuropathy may be further rare in the patients with pSS.

To the best of our knowledge, only four cases of bilateral peripheral facial nerve neuropathy associated with primary Sjögren syndrome have been reported in the literature [7, 8, 9, 10]. These cases, together with our present patient, are summarized in Table 1. All patients were women, with ages ranging from 32 to 83 years. While most presented with typical sicca symptoms, our patient and one previous case showed no overt ocular or oral dryness. Anti‐SS‐A antibody positivity was consistently observed in all cases. Treatment predominantly consisted of corticosteroid‐based regimens, sometimes in combination with other immunosuppressive agents such as methotrexate, intravenous cyclophosphamide, tacrolimus, or intravenous immunoglobulin, and neurological improvement was achieved in most patients. Notably, our patient was the oldest reported to date, highlighting that bilateral facial nerve palsy can be the initial manifestation of pSS even in the absence of prominent sicca features.

**TABLE 1:** Summary of reported cases of bilateral peripheral facial nerve neuropathy in primary Sjögren syndrome, including the present case.

Reference	Age	Sex	Ocular dryness	Oral dryness	SS‐A antibody	SS‐B antibody	Treatment	Outcome
7	69	F	+	+	+	−	mPSL pulse + MTX	+
8	48	F	−	−	+	NA	mPSL pulse + IVCYC	+
9	32	F	+	+	+	NA	PSL	+
10	59	F	+	+	+	+	IVIg + PSL	NA
Present case	83	F	−	−	+	−	PSL + TAC	+

*Note:* “+”: present, “−”: absent.

Abbreviations: IVCYC, intravenous cyclophosphamide; IVIg, intravenous immunoglobulin; mPSL, methylprednisolone; MTX, methotrexate; NA, not available; PSL, prednisolone; TAC, tacrolimus.

Bilateral facial nerve neuropathy is a rare condition also among the general population, being reported in 0.3%–2% cases of facial nerve neuropathy [11]. Of these, Bell's palsy is the most common. Other causes of bilateral facial paralysis include Lyme disease, Möbius syndrome, tumor, bilateral temporal bone fracture, Guillain–Barré syndrome, central nervous system lymphoma, and HIV infection [11]. We ruled out these conditions by the clinical features, serological findings, and imaging examinations.

In our case, diagnosis of pSS was confirmed by the histopathological findings of minor salivary gland biopsy. Xerophthalmia and xerostomia were mild and not enough to diagnose of pSS. Cautious consideration is required in diagnosing pSS with facial paralysis because xerophthalmia and xerostomia can be induced by peripheral neuropathy in the facial nerve via the absence of stimulation to the salivary and lachrymal glands by the facial nerve.

On the other hand, diagnosis of pSS may be difficult without positive immunological or histological findings in the presence of bilateral facial palsy in our speculation. In our case, the positivity of anti SS‐A antibody contributed to the diagnosis of pSS, which was an underlying disease of bilateral facial neuropathy. Serological and histological examinations are rarely performed in clinical practice for the investigation of facial nerve neuropathy.

Overlap between pSS and autoimmune hepatitis has been described in the literature, with reported prevalence ranging from approximately 1% to 4% among patients with pSS [12]. In such cases, autoimmune hepatitis may precede the onset of classical sicca symptoms by several years, as in our patient. Shared autoimmune mechanisms, including B‐cell hyperactivity and the production of disease‐specific autoantibodies, are thought to underlie the coexistence of these two conditions. Recognition of this overlap is important, as liver involvement can remain silent for long periods and may require specific monitoring and management in addition to treatment for pSS‐related manifestations.

In conclusion, we described a rare case of bilateral facial neuropathy complicated by pSS. In the patients with pSS, facial neuropathy is an uncommon complication and further rarely manifests bilaterally. Immunological examinations such as anti‐SS‐A antibody and histological findings of minor salivary glands may contribute to the diagnosis of pSS as a rare underlying disease of bilateral facial palsy.

## Author Contributions

**Ryosuke Hanaoka:** writing – original draft, writing – review and editing.

## Ethics Statement

The author has nothing to report.

## Consent

The patient provided written informed consent to the publication of her personal data.

## Conflicts of Interest

The author declares no conflicts of interest.

## Data Availability

Due to the nature of this case report and privacy concerns, no additional data is available.
